# Condom use across casual and committed relationships: The role of relationship characteristics

**DOI:** 10.1371/journal.pone.0304952

**Published:** 2024-07-18

**Authors:** Cristina A. Godinho, Cícero Roberto Pereira, Ana Pegado, Rita Luz, Maria-João Alvarez

**Affiliations:** 1 NOVA National School of Public Health, Public Health Research Centre, Comprehensive Health Research Center, CHRC, NOVA University Lisbon, Lisbon, Portugal; 2 Instituto de Ciências Sociais, Universidade de Lisboa, Lisboa, Portugal; 3 CICPSI, Faculdade de Psicologia, Universidade de Lisboa, Lisboa, Portugal; University of New South Wales - Kensington Campus: University of New South Wales, AUSTRALIA

## Abstract

**Background:**

While the existence of a complex variety of casual sexual relationships (CSRs) has been acknowledged, studies rarely describe the prevalence of condom use across these relationships or how their hybrid nature, specifically relationship characteristics, affect condom use. This study aims to describe condom use within committed relationships and various types of casual sexual relationships (CSRs), examining the influence of relationship characteristics on condom use among culturally validated relationship types (committed, friends with benefits, hookups, booty call).

**Methods:**

Emerging adults (*N* = 728, 18–29 years, *M* = 22.56; *SD* = 3.01) completed a survey with four blocks: sociodemographics; brief sexual history; relationships over the previous year; and current relationship, assessing relationship type, ten relationship characteristics (e.g., commitment, emotional and sexual exclusivity, partner acquaintance, sexual involvement) and condom use (vaginal, oral, and anal), operationalized by three measures (use at last encounter, likert-type scale and percentage of use).

**Results:**

The results showed patterns in condom use by relationship type and illuminated how relationship characteristics—grouped into three factors: commitment, intimacy, and sexuality—mediate condom use. Condom use was more frequent in vaginal than anal and oral sex, and less frequent in committed relationships. No significant differences were found in condom use in vaginal sex between committed relationships and hookups, with condom in these relationships being significantly lower than in booty call. Intimacy mediated between all contrasts tested and condom use in vaginal sex, while sexuality mediated between committed vs. CSRs and condom use in anal and oral sex.

**Conclusions:**

Findings point to the need of considering the diversity of CSRs for understanding condom use and highlight the role of intimacy as a relevant mechanism associated with condom use in vaginal sex and of sexuality in oral and anal sex, which should be taken into consideration in the tailoring of health promoting efforts.

## Introduction

Sexually transmitted infections (STIs) are a global public health concern, due to its high prevalence and associated morbidity [1], having seen a resurgence in rates in recent years [2, 3]. The World Health Organization estimates that nearly one million people are infected every day with any of the four curable STIs: chlamydia, gonorrhea, syphilis, and trichomoniasis, although this is likely an underestimated value [4–6]. Human Immunodeficiency Virus (HIV) infection is also one of the most common STIs. In 2021 alone, there were approximately 1.5 million new HIV infections and 650,000 deaths attributed to AIDS-related causes [7]. Young adults experience the highest increase in STIs diagnosis rates [8, 9], probably related to more frequent partner changes during this stage of life [10].

Condom use, when used consistently in penetrative practices, is one of the most effective ways to prevent the spread of STIs [11–14], contributing to cost savings in social and healthcare sectors. However, consistent condom use rates tend to be around or below 50% globally [15–17] and have been declining over time [18, 19]. This decline may be explained by the less threatening outcomes of infections, such as those related to HIV, being especially patent among young adults, who have not been exposed to the intense condom promotion efforts made on previous decades [20].

Condom use is primarily observed in casual relationships and with new partners, declining over the duration of the relationship (e.g., [21, 22]), even if the relationship continues to be casual [23]. While the relationship status explains some variation in contraceptive practices, with those in regular relationships being less likely to use condoms and more likely to use hormonal methods, (e.g., [24, 25]), condoms tend to be little used in both regular and casual relationships [18]. Thus, despite a general tendency for greater use in casual when compared to regular relationships, condoms are also used inconsistently and often in less than 50% of occasions in casual relationships [26, 27].

Previous studies have pointed to the existence of longer casual relationships, involving well-known partners, with a variety of emotional manifestations and sexual intimacy [28–31]. Casual sexual relationships (CSRs) proved to be multiple and complex relationships, ranging from one-time, unplanned encounters between strangers, such as *one-night stands*, to ongoing but impersonal and utilitarian encounters, as *booty calls*, to sexual encounters that take place between friends with no intention of becoming partners in a relationship, as in *friends with benefits* (e.g., [31–34]). Different relationship scripts, i.e., the mental representations that guide expectations about the events and sequence of actions in a relationship, have also been found to be distinct across different CSRs [35–37]. *One-night stand* script usually involves flirting, spontaneity, emotional detachment, and a social context frequently involving the consumption of alcohol or other substances and where peers act as facilitators for the encounter, while rules are at the cornerstone of *friends with benefits* and *hookup*, that must be set in order to maintain the uncommitted nature of the relationship, with no obligations involved between the partners, despite the repetition of encounters, preventing the development of romantic feelings [37].

The complex variety of CSRs also involve different relational characteristics, with varying degrees of familiarity and emotional connections in the form of intimacy, passion, and commitment. This is the case among CSR partners who are friends or ex-romantic partners, where different affectionate behaviors (such as cuddling, foreplay, and spending the night) and levels of emotional and sexual exclusiveness and sexual satisfaction may be present [33, 34, 38–40]. Knowledge on the relational processes involved in different types of CSRs is still limited, but the few quantitative studies comparing different CSRs point to relevant differences according to these characteristics (e.g., [37–39]). CSRs proved to be distinct in relation to emotional and sexual involvement, repetition, commitment, exclusivity, premeditation, and partner acquaintance. All of these characteristics are present in *friends with benefits*, are partly present in *hookup* and are almost absent in *one-night stand*, except for sexual involvement [38]. Also, partner familiarity, especially when comparing acquaintances to ex-romantic partners, has been associated with increasing levels of passion, intimacy, and commitment [39]. Taken together, these findings point to the existence of a great heterogeneity in relational characteristics among different types of CSRs, with some showing more similarities than differences to committed relationships that might hamper condom use. However, these studies have been mostly conducted on the socio-emotional implications of the hybrid nature of casual relationships, but not their impact on sexual protection.

Condom use has been shown to be context-dependent, with decisions to use this protection often driven by the nature of the relationship between individual partners [41–44]. An expanding body of research has been shedding light on the importance of relationship characteristics for condom use. High levels of emotional closeness and relationship commitment—but lower levels of intimacy—were associated with reduced condom use and consistency, and increased hormonal method use [45–48]. On the contrary, consistent condom use has been associated with relational variables such as the lower quality of the relationship, lower emotional reasons for sex and lower coital frequency [49, 50]. Nevertheless, these studies explored differences in condom use according to relationship characteristics mainly by contrasting its use in regular relationships in comparison to casual relationships. Also, while these relationship characteristics seem to foster (or hinder) condom use, their putative role in explaining differences in condom use across a range of relationship types is yet to be explored.

Acknowledging the complex variety of CSRs, the present study aims to describe condom use across committed and different types of CSRs, and to expand the knowledge on the association between relationship characteristics and condom use by testing whether these might explain the differences in condom use across different relationship types. More specifically, this study sought to determine condom use in different relationship types by extending the usual committed vs. casual comparison to a variety of casual relationships and to ascertain which relationship characteristics—associated with different relationship types—play a mediating role in condom use.

## Method

### Participants

Initially, 972 respondents participated in the study. Of these, 87 individuals (9.2%) reported never having had sexual intercourse, and another 157 participants did not complete the survey. The final sample consisted of 728 young adults aged 18–29 years (*M* = 22.56; *SD* = 3.01), the majority (67.9%) were women, and a substantial proportion (55.1%) had a college degree.

Before the study, a required sample size of 492 participants was calculated using G*Power to ensure sufficient power to test our predictions. This estimate considered a 95% confidence level and 80% power to detect a statistically significant difference of medium to small effect size (i.e., f = 0.15). Therefore, the current sample size is more powerful than originally thought and allows us to detect smaller effect sizes with reliable results.

### Design and procedure

The present study had a cross-sectional design. A survey was disseminated through email, WhatsApp, and Facebook to contacts from professional groups, faculty members of several Portuguese higher education institutions and personal contacts. Pre-requisites for participation were: (1) to speak European Portuguese as native language, and (2) to have had at least one sexual experience.

Data were collected through *Qualtrics*^®^ platform, from 19^th^ of July 2021 to 19^th^ of January 2022. The questionnaire was pre-tested before the launch of the study to check the flow and to make sure all items were written in a clear language. Participants were informed about the study goals and procedures. Confidentiality and anonymity were ensured, and participants were asked to provide their written informed consent by signing a form before proceeding. All who participated in the study received a gift card of 10€. The study was previously submitted and approved by the Ethics and Deontology Committee of the researchers’ faculty (REF nº 1 2017_18 and REF nº 9 /2021).

### Measures of variables

The questionnaire was composed by four main sections that were presented to all participants: sociodemographic data, brief sexual history, current relationship, and relationships over the previous year (i.e., relationships had in the previous 3 months or in the previous year, if different from the current one).

#### Sociodemographic data

Participants were asked to report their age, gender (men, women, non-binary, other), relational status (single, married, non-marital partnership), religion (e.g., Catholic, Protestant, Orthodox, Islamic), education, and current occupation (student, worker, or student-worker).

#### Brief sexual history

Participants were first asked if they had already been engaged in sexual relations of any sort. When participants mentioned never having had any sexual experience, the questionnaire ended at that point. However, if they reported having already had any sexual contact, a series of questions aiming to describe in more detail the nature of their sexual history were asked, namely: a) age at the first sexual experience, b) number of sexual partners so far, c) gender of those partners (only men, mostly men, men and women, mostly women, only women), and d) participants’ self-identified sexual orientation (heterosexual, homosexual, bisexual, pansexual).

#### Relationships over the previous year

Some questions were included about committed and different types of CSRs that have been selected based on previous studies with Portuguese emerging adults [32, 38], combining qualitative (i.e., association, free recall and evocation tasks) and quantitative methods in order to ascertain which were most representative and culturally-valid. Participants were asked: (1) if they had already been involved in this type of relationships (i.e., committed, hookup, friend with benefits (FWB), booty call, and one-night stand (ONS)), (2) with how many people, (3) whether contraception methods had been used (and which), in relation to committed and CSRs that participants had currently, over the previous 3 months, and over the previous year.

#### Current relationship

Another block of questions asked participants to focus on their current relationship, and on the relationship they had over the previous 3 months and over the previous year, if different from the one they currently had. If participants were involved in more than one relationship at the same time, they were asked to focus on the most significant relationship when answering this block of questions, which included: (a) the type of relationship (i.e., committed, hookup, FWB, booty call and ONS), (b) duration of the relationship, (c) partner gender (male, female, non-binary, other), (d) frequency of intercourse, (e) type of intercourse at the last encounter (i.e., oral, vaginal, anal), (f) whether condom was used (or not) in that encounter. This block also included questions relating to (g) perceived relationship characteristics and (h) two additional measures of condom use that are presented below.

#### Relationship characteristics

For each type of relationship (i.e., committed, hookup, friends with benefits, booty call, and one night stand) that participants mentioned to have or having had in the last 3 months or in the last year, they were asked to rate, in a scale ranging from -3 (*totally absent*) to 3 (*totally present*), the presence of ten relationship characteristics, that were selected on the basis of previous mixed-methods research attesting their relevance and distinctiveness [32, 38, 51]: (a) commitment, (b) emotional exclusivity, (c) sexual exclusivity, (d) emotional involvement, (e) knowledge of the partner, (f) encounter premeditation (defined as “*arranging the encounters in advance*”), (g) intimacy (defined as *“disclosure*, *relaxation*, *sharing personal goals and open and honest communication”*), (h) sexual involvement (with -3 labelled as *“kisses*, *hugs*, *groping”*, 0 labelled as *“oral sex”* and 3 *“penetrative sex”*) and (i) repetition (with labels ranging from -3 *“only one encounter”* to 3 *“more than once a week”*). Besides these characteristics, (j) sexual satisfaction was assessed through five 7-point bipolar scales (i.e., very good-very bad; very pleasant-very unpleasant, very positive-very negative, very satisfying-very unsatisfying, very important-very irrelevant). These showed a good internal consistency and were combined into a single index of sexual satisfaction (Cronbach α = .94).

#### Condom use

According to recommendations [52], condom use was assessed by three different measures. The first was asking whether participants used condom at the last encounter. Second, participants were asked to report condom use for oral, vaginal and anal sex in the last month within each type of relationship they mentioned to have (except for one night stand, as these are not expected to last after the encounter), through a Likert-type scale ranging from 1 (*never*) to 5 (*always*). Last, they were asked to report the number of oral, vaginal, and anal relations they had in the previous month and, of those, how many were condomless. This last measure was then transformed in the percentage of condom use in oral, vaginal, and anal sex. In order to create a single index of condom use for each type of sexual practice (oral, vaginal, and anal), Likert-type measures of condom use and the percentage of condom use were standardized and combined into single indexes of condom use in oral (*r* = .42), vaginal (*r* = .76) and anal (*r* = .63) sex, respectively.

### Data analysis

Data analysis was performed using IBM SPSS. Given that we use both quantitative Likert-type variables and multicategorical measures, it was necessary to use different strategies to analyze the data. For quantitative outcome variables, ANOVA is the better option, while for the multicategorical variables we used the chi-square test to identify significant differences between participants who completed the questionnaire and those who dropped out of the study.

Descriptive statistics and ANOVA were also used to describe participants’ sexual history and current relationships and to test for differences between types of relationships. Repeated measures ANOVA were used to identify significant differences in condom use between sexual practices.

Given the high intercorrelations between relationship characteristics, an exploratory factor analysis (EFA) was conducted in SPSS to examine the structure of variables related to relationship characteristics in CSRs. The factoriability of the CRSs characteristics was acceptable according to Kaiser-Meyer-Olkin (KMO) index (KMO = 0.86) and Bartlett’s test of sphericity, indicating a reasonable correlation between variables (χ^2^ (45) = 1618.35, *p* < .001). Factors were extracted using criteria such as eigenvalues greater than 1 and examination of the scree plot for inflection point. The internal consistency of each factor was assessed using Cronbach’s alpha.

Furthermore, to investigate whether the differences in condom use between the different relationship types could be explained by the three factors identified in the EFA, a total of 12 mediation models were tested using the Open Statistical Software JAMOVI. These models included 4 dummy variables that contrasted: 1) committed relationship vs. CSRs; 2) hookup vs. other CSRs; 3) FWB vs. other CSRs; and 4) booty call vs. other CSRs, with condom use across three sexual practices (oral, vaginal, anal) as dependent variables.

## Results

### Dropout analyses

Considering the initial sample of 972 participants, only gender and age were significantly different between those who finished (*n* = 813) and those who did not complete the survey (*n* = 159), with those who dropped out being slightly older (*M* = 22.92; *SD* = 3.3) than those who completed the study (*M* = 22.34; *SD* = 3.03), *p* = .04, and with those who identified with “other” gender showing a higher dropout (*χ*^2^ = 12.46, *p* = .006). No differences were observed between those who responded and those who dropped out in relation to relational status, religion, education, occupation, whether they already had sexual intercourse (or not), nor in relation to the age at the first sexual intercourse. Hence, those completing the survey were mostly representative of the initial sample.

### Brief sexual history and current relationships

Most participants (67.6%) had their first sexual intercourse between the ages of 15 to 18 years old (*M* = 17.0; *SD* = 2.34; Table 1). No differences in the age at the first intercourse were found for gender, relational status, religion, or education. Only occupation was significantly associated with the age at the first intercourse, with those who worked reporting an older age at the first intercourse (*M* = 17.66; *SD* = 2.81) than those who were students (*M* = 16.81; *SD* = 2.06) or working students (*M* = 16.89; *SD* = 2.51), *p* < .001.

**Table 1:** Sample’s characteristics (n = 728).

Sociodemographic variables		n (%)
Gender	Women	494 (67.9)
	Men	224 (30.8)
	Non-Binary	10 (1.4)
Marital status	Single	667 (93.0)
	Married	16 (2.2)
	Non-marital partnership	35 (4.8)
Religious affiliation	Yes	275 (37.8)
	No	453 (62.2)
Religion	Catholic	250 (34.3)
	Protestant	7 (1.0)
	Orthodox	3 (0.4)
	Islamic	1 (0.1)
	Eastern religions	1 (0.1)
	Other	8 (1.1)
	I prefer not to answer	5 (0.7)
Education	High school or below	327 (44.9)
	Technical school degree (higher education)	83 (11.4)
	Bachelor’s degree	252 (34.6)
	Master’s degree	66 (9.1)
Occupation	Student	442 (60.9)
	Working student	133 (18.3)
	Worker	151 (20.8)
Sexual orientation	Homosexual	43 (5.9)
	Bisexual	65 (8.9)
	Heterosexual	586 (80.5)
	Pansexual	33 (4.7)
Age of first sexual experience	13 or below	25 (3.4)
	14	55 (7.6)
	15	102 (14.0)
	16	136 (18.7)
	17	121 (16.6)
	18	112 (15.4)
	19	57 (7.8)
	20 or over	89 (12.3)
Number of partners	1–2	342 (47.0)
	3–4	153 (21.1)
	5–6	121 (10.8)
	7–8	32 (6.7)
	9 or more	93 (12.7)

The majority of the sample identified as heterosexual (80.6%), followed by bisexual (8.9%), gay/lesbian (5.9%), and pansexual (4.5%). Among women, 84.4% said they had sexual intercourse only with men, 2.8% only with women, and 12.8% with both men and women. Similarly, most men (80.4%) said they only had sexual intercourse with women, with 9.4% reporting having had only with men, and 10.3% with both men and women.

In the number of sexual partners, the distribution was positively asymmetrical (Skewness = 2.06). While the modal category was only one partner (*n* = 222), more than half of the sample mentioned they had at least three partners, with some revealing to having had up to 20 or even more partners (*M* = 4.30; *SD* = 4.47). Most participants (85.7%) reported they already had at least one committed relationship, with the number of committed relationships ranging from one to eight (*M* = 2.04; *SD* = 1.21), and 90.2% of the those who already had a committed relationship having up to three committed relationships. In relation to casual relationships, more than half of the sample (59.1%) mentioned to have already had at least one casual relationship. The distribution of the number of casual relationships was more heterogeneous, with the number of relationships ranging from one to more than 20 (*M* = 4.15; *SD* = 4.35), with 90.2% of the sample who already had a casual relationship reporting having up to eight casual relationships.

More than half of the participants (63.6%) were currently involved in a committed relationship, with most of them (74.3%) being involved in that same relationship for more than 12 months (Table 2). Only a small percentage of participants (10.3%) were currently involved in a CSR, with most being involved in a “friends with benefits” type of CSR. The majority of CSR had been going on for a relative short time (six months or less), but 31.7% of “friends with benefits” and 20% of booty calls lasted more than six months. In relation to partner gender, although heterosexual relationships were the most frequent in all relationship types, the most frequent CSRs among gay men were hookup and booty call, whereas among lesbian the most frequent CSR was friends with benefits.

**Table 2:** Description of relationships.

	Current relationships				Other relationships over the previous year				
	Committed relationship	Hookup	Friends with benefits	Booty call	Committed relationship	Hookup	Friends with benefits	Booty call	One night stand
	463 (63.6%)	18 (2.5%)	41 (5.6%)	15 (2.2%)	119 (16.3%)	83 (11.4%)	83 (11.4%)	45 (6.2%)	48 (6.6%)
**Number of partners**	1–3	1–4	1–6	1–19	1–2	1–19	1–6	1–6	1–9
**Duration**									
Less than 1 day	0 (0%)	0 (0%)	0 (0%)	0 (0%)	0 (0%)	0 (0%)	0 (0%)	0 (0%)	47 (97.9%)
Less than 30 days	7 (1.5%)	6 (37.6%)	10 (24.4%)	6 (40.0%)	9 (7.6%)	60 (72.3%)	30 (36.1%)	33 (73.3%)	1 (2.1%)
1–6 months	60 (12.9%)	6 (37.5%)	18 (43.9%)	6 (40.0%)	29 (24.4%)	23 (27.7%)	38 (45.8%)	11 (24.4%)	0 (0%)
7–12 months	51 (11%)	3 (18.8%)	7 (17.1%)	1 (6.7%)	17 (14.3%)	2 (2.4%)	10 (12%)	1 (2.2%)	0 (0%)
More than 12 months	344 (74.3%)	1 (6.3%)	6 (14.6%)	2 (13.3%)	70 (58.8%)	1 (1.2%)	9 (10.8%)	1 (2.2%)	0 (0%)
**Partner gender**									
Other sex	418 (89.5%)	11 (42.3%)	31 (62%)	13 (52%)	97 (81,5%)	65 (78,3%)	71 (85.5%)	38 (84.4%)	39 (81.3%)
Same sex—men	12 (2.6%)	3 (11.5%)	3 (6%)	2 (8%)	8 (6.7%)	5 (6%)	2 (2.4%)	6 (13.3%)	5 (10.4%)
Same sex—women	21 (4.5%)	2 (7.7%)	4 (8%)	0 (0%)	8 (6.7%)	9 (10.8%)	8 (9.6%)	1 (2.2%)	1 (2.1%)
Other	16 (3.4%)	10 (38.5%)	12 (24%)	10 (40%)	22 (18.5%)	20 (24.1%)	20 (24.1%)	20 (44.4%)	22 (45.8%)
**Frequency of sexual intercourse**									
Never or once in a few months	21 (4.5%)	7 (38.9%)	5 (12.2%)	2 (13.3%)	19 (16%)	40 (48.2%)	18 (21.7%)	15 (33.3%)	NA
1 to 3 times a month	116 (25.1%)	3 (16.7%)	16 (39%)	7 (46.7%)	26 (21.9%)	22 (26.5%)	30 (36.1%)	13 (28.9%)	NA
1–2 times a week	174 (37.6%)	6 (33.3%)	11 (26.8%)	4 (26.7%)	45 (37.8%)	9 (10.8%)	25 (30.1%)	11 (24.4%)	NA
3–4 times a week	93 (20.1%)	0 (0%)	6 (14.6%)	1 (6.7%)	16 (13.4%)	6 (7.2%)	8 (9.6%)	3 (6.7%)	NA
5 or more times a week	45 (9.7%)	0 (0%)	2 (4.9%)	0 (0%)	9 (7.6%)	5 (6%)	5 (6%)	1 (2.2%)	NA
More than once a day	14 (3%)	2 (11.1%)	1 (2.4%)	1 (6.7%)	8 (6.7%)	3 (3.6%)	0 (0%)	2 (4.4%)	NA
**Contraception method** ^a^									
Withdrawal	81 (17.5%)	2 (11.1%)	3 (7.3%)	3 (20.0%)	18 (15.1%)	4 (4.8%)	12 (14.5%)	6 (13.3%)	4 (8.3%)
Natural methods (e.g. temperature)	8 (1.7%)	0 (0%)	0 (0%)	0 (0%)	1 (0.8%)	0 (0%)	1 (1.2%)	0 (0%)	0 (0%)
Contraceptive pill	245 (52.9%)	5 (27.8%)	14 (34.1%)	4 (26.7%)	44 (37%)	16 (19.3%)	31 (37.4%)	17 (37.8%)	9 (18.8%)
Other hormonal (e.g., ring)	35 (7.6%)	0 (0%)	3 (7.3%)	2 (13.3%)	4 (3.4%)	1 (1.2%)	4 (4.8%)	3 (6.7%)	1 (2.1%)
Intrauterine device	15 (3.2%)	1 (5.6%)	2 (4.9%)	2 (13.3%)	4 (3.4%)	3 (3.6%)	4 (4.8%)	1 (2.2%)	2 (4.2%)
Male condom	184 (39.7%)	11 (61.1%)	21 (51.2%)	9 (60.0%)	44 (37.0%)	33 (39.8%)	41 (49.4%)	31 (68.9%)	31 (64.6%)
Female condom	1 (0.2%)	0 (0%)	0 (0%)	0 (0%)	0 (0%)	0 (0%)	0 (0%)	0 (0%)	0 (0%)
**Sexual practices- last encounter**									
Oral sex	398 (86%)	13 (72.2%)	33 (80.5%)	13 (86.7%)	108 (90.8%)	47 (56.6%)	67 (80.7%)	41 (91.1%)	37 (77.1%)
Vaginal sex	419 (90.5%)	9 (50%)	33 (80.5%)	10 (66.7%)	101 (84.9%)	49 (59%)	70 (84.3%)	36 (80%)	38 (79.2%)
Anal sex	108 (23.3%)	5 (27.8%)	8 (19.5%)	2 (13.3%)	25 (21%)	8 (9.6%)	12 (14.5%)	9 (20%)	7 (14.6%)
**Condom used- last encounter** ^b^									
Oral sex	12 (3%)	2 (15.4%)	2 (6.1%)	1 (7.7%)	4 (3.4%)	5 (6%)	2 (2.4%)	1 (2.2%)	1 (2.1%)
Vaginal sex	207 (49.4%)	6 (66.7%)	21 (63.6%)	5 (50%)	45 (37.8%)	24 (28.9%)	46 (55.4%)	27 (60%)	26 (54.2%)
Anal sex	45 (41.7%)	2 (40%)	2 (25%)	2 (100%)	8 (6.7%)	4 (4.8%)	7 (8.4%)	5 (11.1%)	4 (8.3%)

Note.

^a^ The frequencies presented represent those that, for each type of relationship, have reported to “very often” or “always” use a given contraceptive method.

^b^ The frequencies reported are for those who said to have had each of the sexual practices.

NA = not applicable.

The sexual practices most reported at the last encounter were oral and vaginal sex. Oral sex was more frequently reported than vaginal sex in hookup and booty call, equally frequent in friends with benefits, whereas vaginal sex was more frequently reported than oral sex in committed relationships. As for contraceptive methods, the contraceptive pill was the most frequently used in committed relationships, while male condom was the most frequently used method in all CSRs.

### Condom use

**Fig 1:**
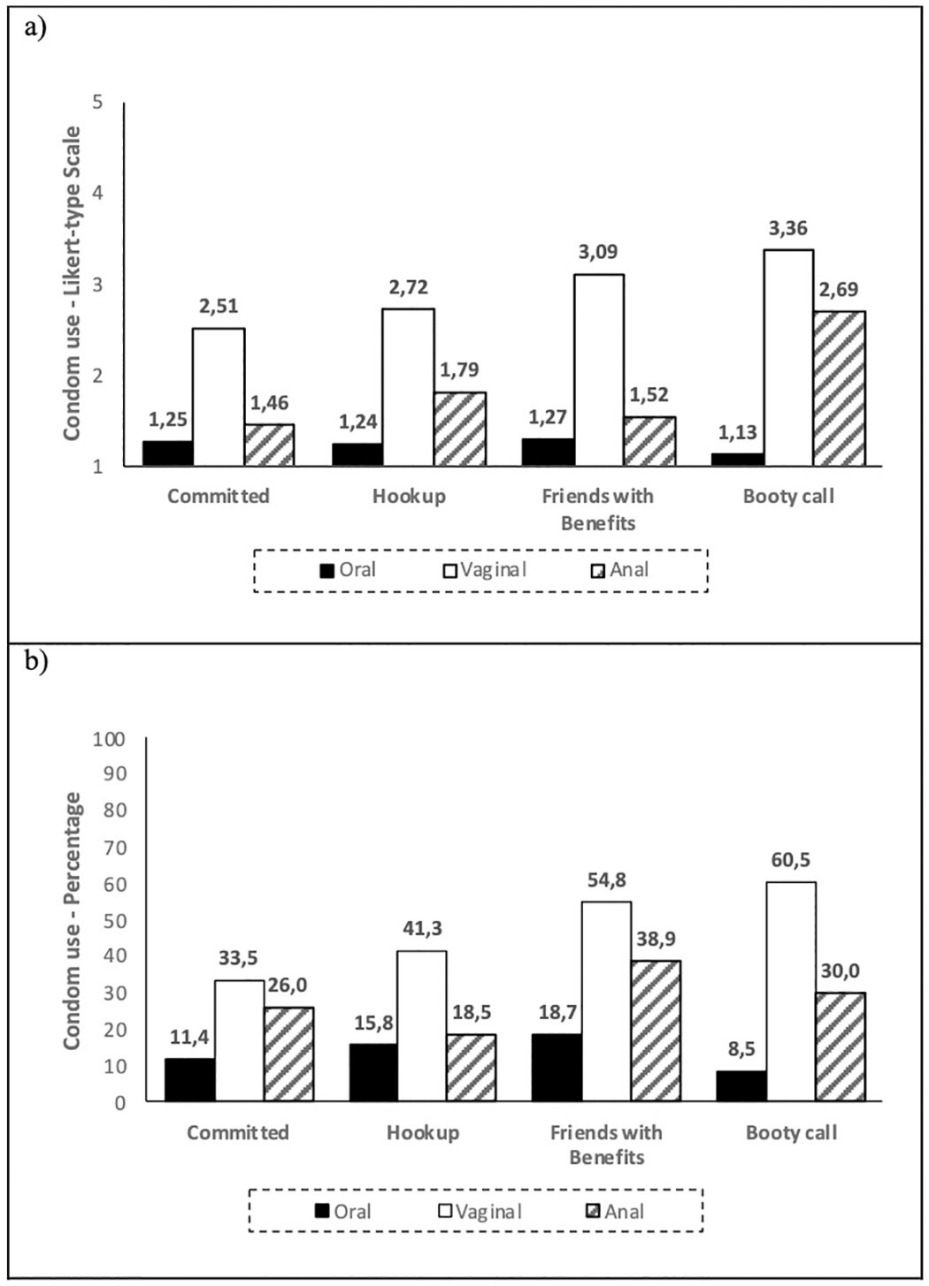
Condom use for different sexual practices in different types of relationships, assessed by (a) a 5-points likert-type scale and by (b) percentage of use.

Overall, condom use was less reported in oral sex than in the other practices (Fig 1). When assessed by the percentage of use in relation to the total number of encounters where a specific sexual practice occurred, condom use in oral sex was reported to be used, on average, only on 12.6% of sexual encounters. The figure rose to 27.5% for sexual encounters involving anal sex, and to 36.7% of sexual encounters involving vaginal sex. Likewise, condom assessed through the Likert-type scale use was lower for oral intercourse (*n* = 764; *M* = 1.24; *SD* = 0.85), followed by anal intercourse (*n* = 364; *M* = 1.55; *SD* = 1.32) and higher in vaginal intercourse (*n* = 771; *M* = 2.66; *SD* = 1.77). Repeated measures ANOVA also showed significant within-subject differences (*n* = 319) in condom use across the three types of sexual practices, again with lower condom use for oral sex (*M* = 1.20; *SE* = 0.04), followed by anal sex (*M* = 1.46; *SE* = 0.07), and higher use for vaginal sex (*M* = 1.93; *SE* = 0.08), all *p*’s < 0.001.

Condom use in vaginal intercourse was significantly lower in committed relationships than in booty call, and friends with benefits when assessed by percentage of use, but no significant differences were found in relation to hookup, or friends with benefits when assessed by Likert-type scale (Table 3). For anal intercourse, condom use (assessed by Likert-type scale) was significantly higher in booty call when compared to the other relationship types. For oral sex, no significant differences were found in condom use across the different relationship types.

**Table 3:** Condom use for different sexual practices in different types of relationships.

		Committed Mean (*SD*)	Hookup Mean (*SD*)	Friends with benefits Mean (*SD*)	Booty call Mean (*SD*)	*F*	*p-value*
**Likert-type scale**	**Oral**	1.25 (0.84)	1.24 (0.88)	1.27 (0.96)	1.13 (0.68)	0.35	.79
	**Vaginal**	2.51 (1.73)^a^	2.72 (1.79)^a,b^	3.09 (1.81)^a,b^	3.36 (1.76)^b^	6.05***	< .001
	**Anal**	1.46 (1.20)^a^	1.79 (1.59)^a^	1.52 (1.28)^a^	2.69 (1.99)^b^	4.94**	< .01
**Percentage**	**Oral**	11.4 (30.6)	15.8 (34.7)	18.7 (39.0)	8.5 (28.2)	1.80	.15
	**Vaginal**	33.5 (44.0)^a^	41.3 (47.1)^a,b^	54.8 (46.5)^b,c^	60.5 (45.6)^c^	9.83***	< .001
	**Anal**	26.0 (42.7)	18.5 (38.6)	38.9 (50.2)	30.0 (43.6)	0.63	.60
**Standardized index**	**Oral**	-0.02 (0.83)	0.04 (0.86)	0.11 (1.03)	-0.06 (0.88)	0.83	.48
	**Vaginal**	-0.11 (0.92)^a^	-0.01 (0.91)^a^	0.25 (0.98)^a,b^	0.41 (1.00)^b^	8.07***	< .001
	**Anal**	-0.15 (0.78)	-0,04 (0.87)	-0.04 (0.86)	0.38 (1.06)	2.29	.08

*Note*. Means with different superscripts are significantly different at *p* < .05.

### Relationship characteristics

**Fig 2:**
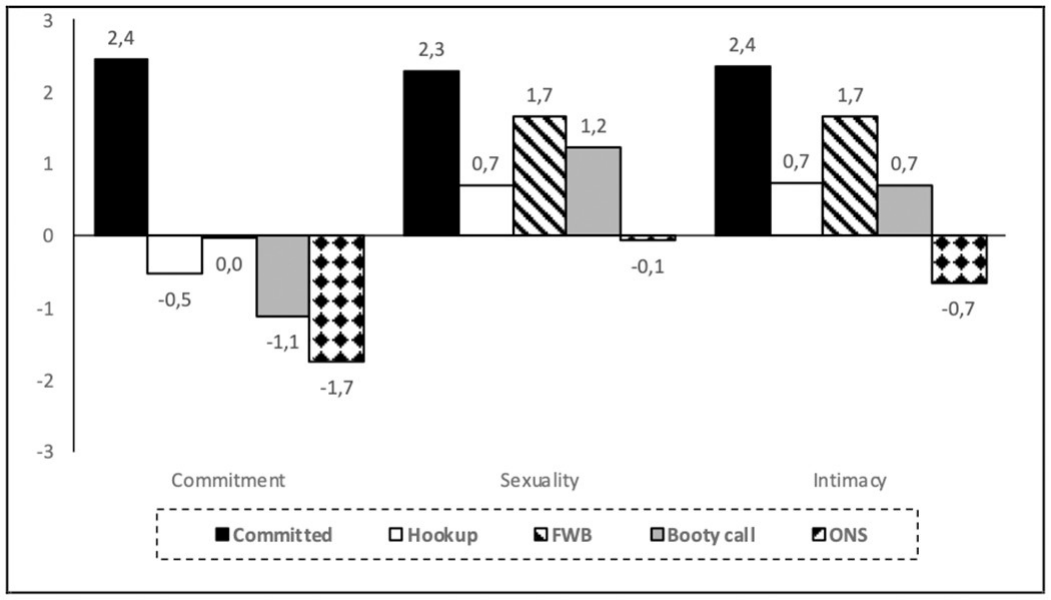
Levels of commitment, sexuality and intimacy in committed and casual sexual relationships. *Note*. FWB = friends with benefits; ONS = one-night stand.

All ten analyzed relationship characteristics were deemed to be more present in committed relationships than in casual relationships, except for sexual involvement (that was similar to friends with benefits and booty call relationships) and repetition (that was similar to friends with benefits) (Table 4). Overall, casual relationships were rated as involving low levels of commitment, as well as low levels of emotional, and sexual exclusivity. However, friends with benefits and hookup were characterized by a greater emotional involvement and higher knowledge of the partner than booty call and one night stand (see Fig 2).

**Table 4:** Characteristics of different types of relationships.

	Committed Mean (*SD*)	Hookup Mean (*SD*)	Friends with benefits Mean (*SD*)	Booty call Mean (*SD*)	One night stand Mean (*SD*)	*F*	*p-value*
**Commitment**	2.66 (0.94)^a^	-0.81 (1.97)^b,c^	-0.32 (2.02)^b^	-1.28 (1.94)^c^	-1.96 (1.74)^d^	370.25	< .001
**Emotional exclusivity**	2.21 (1.47)^a^	-0.79 (2.01)^b,c^	-0.34 (1.99)^b^	-1.41 (1.87)^c,d^	-2.06 (1.59)^d^	209.59	< .001
**Sexual exclusivity**	2.33 (1.57)^a^	-0.46 (2.19)^b^	0.15 (2.24)^b^	-1.00 (2.13)^c^	-1.46 (2.10)^d^	139.31	< .001
**Emotional involvement**	2.58 (0.97)^a^	0.01 (2.00)^b^	0.45 (1.86)^b^	-0.74 (2.20)^c^	-1.48 (1.75)^d^	230.78	< .001
**Knowledge of the partner**	2.55 (0.96)^a^	1.21 (1.64)^b^	1.81 (1.39)^c^	0.48 (2.09)^d^	-0.56 (2.09)^e^	109.23	< .001
**Encounter premeditation**	1.87 (1.50)^a^	0.35 (2.14)^b^	1.34 (1.85)^c^	1.20 (1.79)^b^	-0.92 (2.06)^d^	45.26	< .001
**Intimacy**	2.64 (0.91)^a^	0.66 (1.88)^b^	1.87 (1.35)^c^	0.41 (2.04)^b^	-0.48 (2.04)^d^	136.39	< .001
**Sexual involvement**	2.48 (1.19)^a^	0.72 (2.06)^b^	1.97 (1.56)^a^	2.03 (1.60)^a^	1.31 (1.70)^c^	40.23	< .001
**Repetition**	2.10 (1.44)^a^	0.46 (2.08)^b^	1.44 (1.65)^a^	0.44 (2.25)^b^	-1.42 (2.09)^c^	74.72	< .001
**Sexual satisfaction**	2.43 (1.25)^a^	0.79 (1.60)^b^	1.52 (1.30)^a,b^	0.71 (2.30)^b^	-0.67 (1.97)^c^	31.77	< .001

*Note*. Means with different superscripts are significantly different at *p* < .05.

For casual relationships, the analyzed relationship characteristics clustered around three factors, explaining 70.8% of the variance (see S1 Table). Only the variable “sexual satisfaction” had a communality below 0.40, indicating that this variable has a lower contribution to the explained variance. The first factor, that was labelled as “commitment” (Cronbach α = 0.93) was composed by emotional exclusivity, commitment, sexual exclusivity, and emotional involvement; the second factor, that was labelled as “sexuality” (Cronbach α = 0.70) was composed by sexual involvement, repetition, and sexual satisfaction; the last factor, that was labelled as “intimacy” (Cronbach α = 0.79), was composed by intimacy, partner acquaintance, and premeditation.

All casual relationships were rated as entailing low levels of commitment (Fig 2). Notwithstanding, significant differences in commitment levels were also found between them, with one-night stand (ONS) showing the lower levels of commitment, followed by booty call, and only after by hookup and friends with benefits (FWB), that were rated as having the same commitment level. In relation to sexuality, significant differences were found between all relationship types (except between booty call and FWB), with ratings being lower for ONS, followed by hookup, then by booty call and FWB, and finally by committed relationships. For intimacy, significant differences were found between all relationship types (except between booty call and hookup), with ratings being lower for ONS, followed by booty call and hookup, then by friend with benefits and finally for committed relationships.

### The mediating role of relationships characteristics in condom use

**Fig 3:**
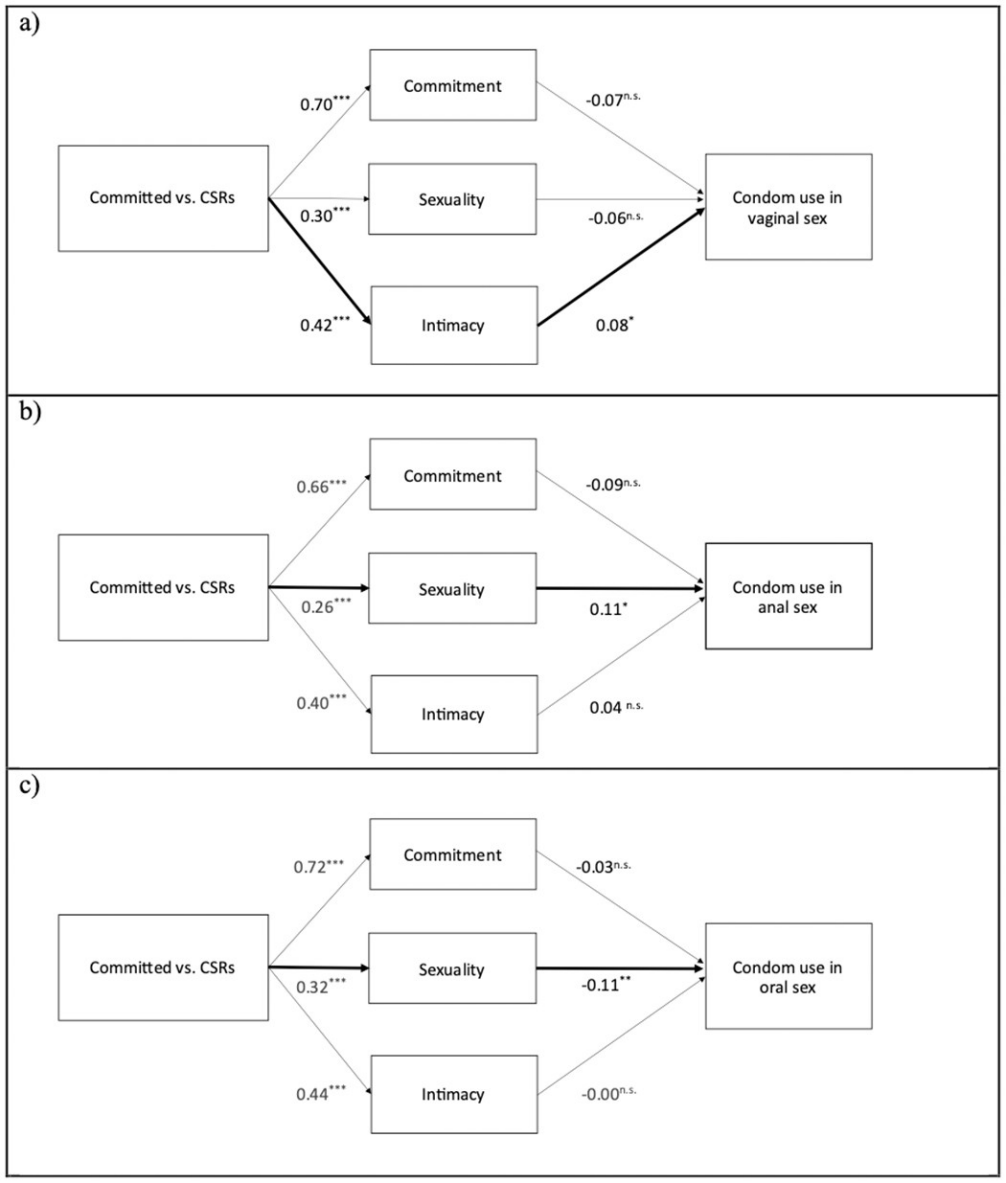
Mediation models of condom in use in committed vs. casual sexual relationships for vaginal (a), anal (b) and oral sex (c). *p < 0.05; **p < 0.01; *p < 0.001.

When comparing committed relationships with CSRs, significant mediation pathways were observed for all three sexual practices. Differences in condom use between committed relationships and CSRs were found to be mediated by the degree of intimacy during vaginal sex (β_indirect effect_ = 0.03, *p* = .04). Specifically, individuals in committed relationships reported higher levels of intimacy, and the more intimacy they reported, the more condoms they used (Fig 3a). Moreover, differences in condom use were mediated by sexuality for both anal (β_indirect effect_ = 0.03, *p* = .05) and oral sex (β_indirect effect_ = -0.03, *p* < .01), albeit in opposite directions. Specifically, individuals in committed relationships reported higher levels of sexuality (i.e., sexual engagement, repetition, and sexual satisfaction), which was associated with increased condom use during anal sex (Fig 3b) and decreased condom use during oral sex (Fig 3c).

**Fig 4:**
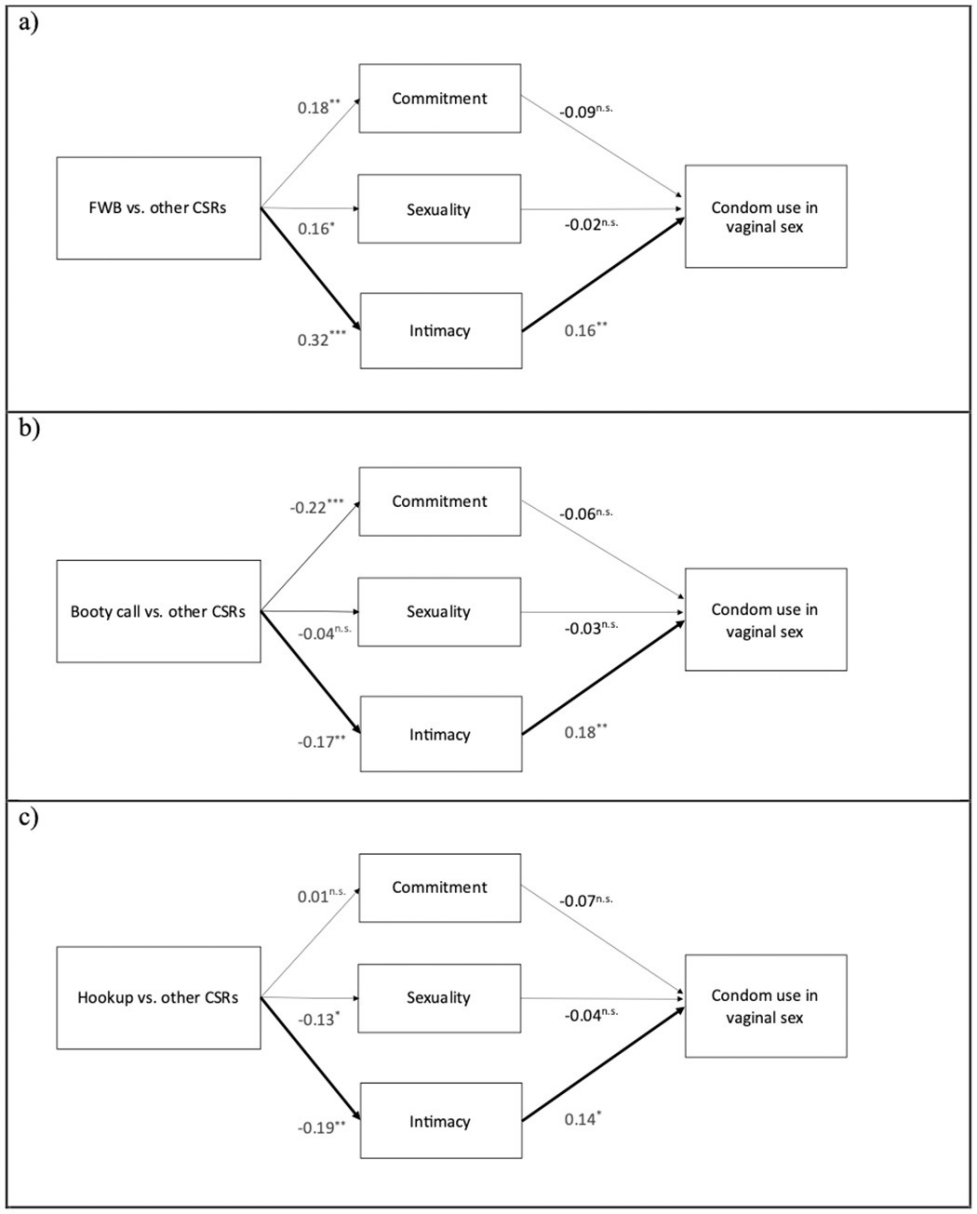
Mediation models of condom in use in friends with benefits (a), booty call (b) and hookup (c), versus the other CSRs, for vaginal sex. *p < 0.05; **p < 0.01; *p < 0.001.

When comparing each type of CSR with other CSRs, significant mediation pathways were found only for vaginal sex, but not for anal or oral practices. The role of relationship type on condom use during vaginal intercourse was significantly mediated by intimacy when comparing FWB to other CSRs (β_indirect effect_ = 0.05, *p* = .03; Fig 4a), with individuals in FWB reporting more intimacy associated with more condom use. Regarding the comparison of booty call with other CSRs (β_indirect effect_ = -0.03, *p* = .05; Fig 4b), the mediation analysis showed a different pattern of effects, as while intimacy was associated with increased condom use, individuals in booty call relationships reported less intimacy in such relationships. A similar pattern emerged with marginal significance when comparing hookup with other CSRs (β_indirect effect_ = -0.03, *p* = .08; Fig 4c).

## Discussion

The complexity and hybrid nature of CSRs has been acknowledged in recent years, bringing about a fuller understanding of their spectrum, the specific context in which these encounters occur, and their specific characteristics. Even so, variables pertaining to the relationship characteristics had not yet been explored as potential mechanisms to explain differences in condom use across relationship types. This study aimed to go beyond the distinction between casual sex and sexual encounters occurring within the context of committed relationships, and tested whether a set of previously studied relationship characteristics could explain condom use in different sexual practices (oral, vaginal, and anal sex) across culturally validated relationship types. Another goal was to determine condom use associated with different relationship types, characteristics, and practices.

Overall, our results point to condom use in emerging adults being more frequent in vaginal sex, particularly in the context of booty call relationships. No significant differences in condom use in vaginal sex between committed relationships and hookup (and with friends with benefits, when assessed by a Likert-type scale) were observed, attesting more similarities than differences across relationship types in condom use. Importantly, for all studied relationships, the level of intimacy between partners played a critical role in determining condom use. Higher levels of intimacy, including partner acquaintance and premeditation, were associated to more frequent condom use in all casual relationships, as well as is in committed relationships. Affectionate behaviors, considered aspects of intimacy, were found in casual sexual encounters [33], with more familiar CSRs partners offering higher levels of intimacy [34, 39]. Thus, as found in other studies, the findings of the present study concur to CSR not being intimacy-free narratives. More than the CSR in which people consider themselves to be involved, it is the way CSR are characterized and the type of sexual practice that seems to determine condom use.

These findings are also in line with the communication model [22, 45], that sustains that contraceptive use is higher with increasing levels of knowledge and intimacy between partners. In fact, when there is more intimacy, sexual encounters tend to be more planned, allowing for the enaction of preparatory behaviors, such as buying condoms and carrying them, which are relevant self-regulatory processes that operate between motivations to use condoms and actual behavior [53]. More intimacy can also mean more openness for discussions around contraception and condom use, stressing the relevance of communication in supporting decisions around condom use [54, 55]. In this vein, our results highlight the importance of interventions reinforcing communication skills about condom use with emerging adults and to make condoms widely available, especially in places where unplanned sexual encounters tend to occur among emerging adults, as not having a condom at hand at the time of sexual intercourse is often cited as a barrier for lack of use (e.g., [56]). Although sexual education has been mandatory in the Portuguese education system since 2009 for all children over the age of 6, there is still an emphasis on a biological-hygienist approach primarily centered around biology and the potential risks of sexual behavior, similar to other countries [57–59]. This hygienist perspective pays some attention to condom use but overlooks the multiple relational contexts in which sexual exchanges occur and completely neglects casual sexual relationships, failing to meet the objective of addressing topics that have become a priority in recent years [60, 61]. By law, young people, including young adults, have access to free family planning services in the National Health Service, which includes access to free contraceptives to prevent sexually transmitted infections (STIs) and pregnancy. However, accessing consultations may not be easy, and very few or no condoms are provided. Relatedly, it is important to stress that our study did not assess how young adults acquire condoms nor make decisions regarding the use of protection with their partner(s), which would be an important avenue for future research.

Condom use was also more frequent in vaginal sex than in oral or anal sex, which can be related to the motivation to use condoms in heterosexual relationships revolving more around avoiding pregnancy than avoiding STIs. Previous research has shown that adolescent risk perception on STIs tends to be low [62–64], with the use of condoms being mostly a backup for pregnancy prevention than STI prevention [65, 66]. Interestingly, it was sexuality (and not intimacy) that was found to mediate between relationship type and condom use for anal and oral sex, possibly tied to a greater focus on sexual exploration and pleasure attached to these sexual practices, although with opposite effects. Whereas higher levels of sexuality—namely sexual involvement, repetition and sexual satisfaction -, were associated with increased condom use in anal sex, they were associated with lower condom use in the case of oral sex. Although more research is needed to better understand the motivations and patterns of engagement in protected oral and anal sex in this segment of the population, previous research points to condomless oral sex being perceived as less risky for STIs among the youth [67], while condomless anal sex with a casual partner being perceived as highly risky [68]. It could be the case then that higher exposure, through repetition of encounters and higher sexual involvement, tends to favor its use in anal sex where risk is perceived as high, but tends to decline in oral sex, where risk is perceived as low. Either way, considering the role of sexuality in condom use for oral and anal sex, promotion efforts should also focus on the benefits of condom use attached to sexual pleasure.

Sexual practices also varied across the different relationship types. Although overall oral and vaginal sex are the most common practices, vaginal sex was more frequent in the context of committed relationships, while oral sex was most frequently reported in the different casual relationships. Recent studies on hookups have also found that non-coital sexual behaviors are the most frequent in these relationships [69, 70], while vaginal sex is a more representative behavior in “friends with benefits” [71, 72] than in situations with someone known for less than 24 hours [73]. This may reveal oral practices as mainly focused on obtaining pleasure and as a means of sexual exploration, while vaginal sex is attached to higher levels of intimacy and commitment in relationships, sometimes also associated with romantic views of these relationships [74, 75].

Contraception methods also tended to vary across relationship types. Contraceptive pill was the most frequently used method by those in committed relationships in our sample and in keeping with the hypothesis derived from the sawtooth pattern [48], in which condom tends to be present at the beginning of a relationship and declines thereafter, reappearing at the start of another relationship and again declining over time. As the relationships lengthen and become more serious condom use declines and partners tend to transition to hormonal methods. This result may in part also be related to greater preponderance of vaginal sex in these relationships and hence, as previously mentioned, a greater focus on preventing undesired pregnancies rather than STIs. Also, the greater frequency and premeditation of sexual encounters in committed relationships—not to mention condom use being perceived as a lack of trust [76]–allows to opt for contraceptive methods that require taking them in advance, as is the case with the contraceptive pill. In contrast, male condom was the most used contraceptive method in CSRs. Not only is it a more suitable method for rather unpremeditated encounters, where repetition may not occur, but also because these sexual encounters may imply higher risks, in part associated to the non-exclusive nature of those relationships. In fact, protection against STIs tends to be a more relevant driver for condom use in CSRs than pregnancy concerns [77].

Regarding relationship characteristics, the ten that were considered in this study were found to cluster, through an exploratory factor analysis, around three factors. Those factors are similar to those presented in Sternberg’s triangular theory of love [78]. In this theory, different types of intimate relationships can be characterized through the combination of three factors: a) *passion*, referring to “the drives that lead to romance, physical attraction, sexual consummation and related phenomena” (p.119), that in our study corresponded to the “sexuality” factor, integrating sexual involvement, repetition and, to a lesser extent, sexual satisfaction; b) *intimacy*, referring to “feelings of closeness, connectedness, and bondedness” (p.119), that in our study corresponded to the factor that was labelled under the same name and which included ratings of intimacy, partner acquaintance, and premeditation; c) *commitment*, which is “in the short term, the decision that one loves someone else, and in the long term, the commitment to maintain that love” (p. 119), and which was composed by ratings of emotional exclusivity, commitment, sexual exclusivity and emotional involvement in our study. Sternberg’s theory had already been used to describe different types of relationships, including steady [79], and different types of casual relationships [39, 80]. In our study, the three factors allowed for richer and granulated distinctions between the different relationship types. In line with results found by Rodrigue and colleagues [39], also with a sample of emerging adults, in our study casual relationships entailed low levels of commitment, expressed by lower exclusivity and emotional involvement. This is consistent with the views of CSRs as being more focused on sexuality and less on the development of emotional bonds when compared to committed relationships. Nevertheless, intimacy and sexuality were present in all CSRs (except for one-night stand), which is not surprising when a significant percentage of casual encounters (25%) last for more than six months, with some lasting for one or more years, differing from what in the theory configures the “romantic love”. Therefore, our results provide support to the notion that some types of CSRs include emotional connections in the form of intimacy between partners, especially in the case of “friends with benefits”, where although not definitional to the relationship, an implicit notion of commitment, in the form of emotional involvement and sexual exclusivity may even be present [33, 39, 81].

Taken together, our results align with emerging adults still reporting a preference for romantic, in addition to casual, sexual relationships [82, 83]. Although slightly more than half of participants (59.1%) mentioned they already had at least one casual relationship, committed relationships were more prevalent (85.7%). Moreover, most current relationships or relationships held over the year before the survey were identified by participants as being committed type of relationships, with most of these being long-term (i.e., with a duration of more than 1 year). These results also align with those from previous studies conducted with emerging adults from the same cultural background (e.g., [37, 38]), which did not find evidence for a *hookup culture* [84, 85], a phenomenon that has been identified mostly in the campuses of North American universities, configurating a change in relational practices associated with a decline in committed type of relationships and an increase in casual sexual encounters, that became normative [86, 87]. This calls attention to the relevance of studying relational and sexual practices in different cultures and for the need of interventions in this area to be culturally sensitive. Nonetheless, the total number of those who mentioned have been enrolled in a casual relationship was nearly twice than the figures presented by previous studies with Portuguese university students [88, 89], which may indicate that casual relationships are also on the rise. In contrast, the age at first intercourse found in the present study is in line with the results of previous studies with national representative samples [15, 90].

Almost one in six participants in our sample identified as gay/lesbian, bisexual or pansexual. Interestingly, there was a tendency for gay CSR to be those where lower levels of commitment and intimacy were reported, such as hookup and booty call, while the FWB relationship type, in which these characteristics are more present, was more frequent in lesbian CSR. This may be a sign of the pervasiveness of a sexual double standard in how people in same sex relationships approach these relationships, in the form of a differential awareness of stigma regarding the involvement in CSRs [91]. On the other hand, most of same-sex relationships tended to occur in the context of one-night stands, which may reflect a tendency for these to occur occasionally, and in a more unplanned way. It is important to highlight, however, that future studies are needed in order to draw firmer conclusions on the role of partner gender and sexual orientation on the nature of relationships and, consequently, on the use of condoms within those relationships, considering the relatively small number of non-heterosexual relationships that were reported by our participants.

This study had two main limitations that should be acknowledged. The first is the reliance on self-report measures to estimate condom use across different relationships, which may be subject to recall and social desirability bias. In fact, the different self-reported measures that were used to assess condom use point to big differences in prevalence. When assessed by use at the last encounter, the percentage of use found for vaginal sex is similar to previous studies (i.e., around 50%;[15, 16]). However, when evaluated by the percentage of use, the figures tend to drop both for vaginal and anal sex (i.e., 50.7% vs. 47.5% for vaginal sex; 41.5% vs. 28.35% for anal sex), and increase for oral sex (i.e., 3.7% vs. 13.6%). Although measuring condom use at last sexual encounter tends to be the most used measure for condom use, as it is easy to understand and minimizes potential for recall bias [52], some have expressed concerns that it might not be adequate to estimate STIs risk as it measures behavior in only one sexual encounter. It would be important to assess, using more objective measures, such as those used in biomarkers validation studies (e.g., [92]), which of the used self-report indicators is more reliable. Nevertheless, evidence has shown that using a “test-retest” strategy, using more than one differently worded questions on condom use, such as the one used in the present study, can help to correct for overreporting associated with recall bias and social desirability [93]. Therefore, we contend that the index of condom use that was used in this study may have helped to minimize bias associated with the self-reported nature of measures used.

Another limitation is that the mediations were tested with cross-sectional data. Ideally, the outcome measure (i.e., condom use) should not have been assessed at the same point in time than the independent variable (i.e., relationship type) and mediators (i.e., relationship characteristics). Although the study had been designed as a longitudinal sample, with two data collection moments (baseline and one-month), it was not feasible to use the longitudinal data due to different reasons: mortality in the sample; some CSRs not being very frequent and others ending within the study time, making it inviable to assess condom use at a later time and; some sexual practices, such as anal sex, not being very frequent in the context of some CSRs. Hence, causal inferences are not warranted, nor the direction of the influences between studied variables.

## Conclusions

Our study expands the previous literature by analyzing condom use within the context of different casual relationships and sexual practices, using partner-specific measures instead of individual measures, with different operationalizations of condom use, and by inspecting the relational mechanisms that underlie sexual protection. In line with the literature, our findings point to condom use being more frequent in vaginal sex than anal or oral sex and being less frequent in committed relationships. Importantly, the findings also showed that condom use in some types of CSRs, particularly in hookups and friends with benefits, does not differ significantly from committed relationships and that relevant differences in condom use across CSRs exist, namely between hookup and booty call, being significantly lower in the context of the former.

Taken together, this points to a more granulated outlook of sexual relationships among emerging adults, with relevant implications for social policies and sexual health interventions for the promotion of sexual protection. First, there is the need to ensure the inclusion of the topic of different types of relationships in the sexual education of young people to raise awareness of the nuances in various CSRs, especially the characteristics of some that hinder condom use, even in occasional contexts. Secondly, our study also contributes with identifying clusters of relationship characteristics that are relevant for condom use in different sexual practices across different types of committed and casual relationships, particularly stressing the role of intimacy between partners as a mechanism associated with condom use in vaginal sex and the role of sexuality in condom use in oral and anal sex, which is crucial for adapting interventions to individual and contextual characteristics. Lastly, knowledge on the emotional and sexual characteristics of different CSRs can be used to increase communication between partners, enabling them to better define the sexual situations they are involved in and decreasing unwanted experiences. These proposals have the potential to help understand the dynamics associated with condom use in different CSRs, increase their use, and improve individuals’ sexual health.

## Supporting information

S1 Table Summary of exploratory factor analysis. (DOCX)

S1 Appendix Dataset used in the reported analyses. (SAV)
