# Supplementary material for: Condom use across casual and committed relationships: The role of relationship characteristics
Source: PLoS One. 2024 Jul 18;19(7):e0304952. doi: 10.1371/journal.pone.0304952 (PMC11257321; doi:10.1371/journal.pone.0304952)
Supplement: S1 Table — (DOCX) [file pone.0304952.s001.docx]

S1 Table – Summary of exploratory factor analysis

|  | **Factor loadings after rotation** | | |
| --- | --- | --- | --- |
| **Variables** | Commitment | Sexuality | Intimacy |
| emotional exclusivity | 0.958 |  |  |
| commitment | 0.822 |  |  |
| sexual exclusivity | 0.747 |  |  |
| emotional involvement | 0.558 |  |  |
| sexual involvement |  | 0.822 |  |
| repetition |  | 0.441 |  |
| sexual satisfaction |  | 0.315 |  |
| intimacy |  |  | -0.910 |
| partner acquaintance |  |  | -0.725 |
| premeditation |  |  | -0.524 |
| Eigenvalues | 3.613 | 1.692 | 3.254 |
| % variance explained | 42.651 | 9.348 | 7.763 |
